# Active Microwave Metamaterials Incorporating IdealGain Devices

**DOI:** 10.3390/ma4010073

**Published:** 2010-12-29

**Authors:** Li-Ming Si, Tao Jiang, Kihun Chang, Te-Chuan Chen, Xin Lv, Lixin Ran, Hao Xin

**Affiliations:** 1 Department of Electronic Engineering, School of Information and Electronics, Beijing Institute of Technology, Beijing 10081, China; E-Mails: siliming@email.arizona.edu (L.M.S.); lvxin@bit.edu.cn (X.L.); 2 Department of Information Science and Electronic Engineering, Zhejiang University, Hangzhou 310027, China; E-Mails: jtzju@126.com (T.J.); ranlx@zju.edu.cn (L.R.); 3 Electrical and Computer Engineering Department, University of Arizona, Tucson, AZ 85721, USA; E-Mail: changkh@ece.arizona.edu (K.C.)

**Keywords:** active metamaterial, negative resistance device, composite right/left-handed transmission line (CRLH-TL), dispersion/attenuation relation

## Abstract

Incorporation of active devices/media such as transistors for microwave and gain media for optics may be very attractive for enabling desired low loss and broadband metamaterials. Such metamaterials can even have gain which may very well lead to new and exciting physical phenomena. We investigate microwave composite right/left-handed transmission lines (CRLH-TL) incorporating ideal gain devices such as constant negative resistance. With realistic lumped element values, we have shown that the negative phase constant of this kind of transmission lines is maintained (*i.e.*, left-handedness kept) while gain can be obtained (negative attenuation constant of transmission line) simultaneously. Possible implementation and challenging issues of the proposed active CRLH-TL are also discussed.

## 1. Introduction

Metamaterials are artificial composite materials that can provide unique and very useful properties such as negative index of refraction, which do not normally exist in natural material, and may lead to many new and exciting capabilities for electromagnetic applications. Metamaterial based microwave circuits and antennas may be ideal for future communication and sensing systems. However, many of the practical applications are limited by two fundamental issues that have not yet been solved, namely, loss and narrow bandwidth inherently associated with a typical metamaterial. The key for future breakthrough is to understand these fundamental limitations and identify effective solutions for realizing low loss and broadband metamaterials that are critical for many of the proposed and potential applications.

Transmission line based metamaterials at microwave frequencies, such as composite right/left-handed transmission line (CRLH-TL) with distributed series capacitance and shunt inductance loading in addition to a conventional right-handed line [1,2,3], is an alternative approach for implementing left-handed metamaterials (LHM), which have the advantages of simple structure, relatively low loss and broad bandwidth compared to resonant-type metamaterials. Such metamaterials have been used in designs of antennas, filters, couplers, power dividers, phase shifters, sub-wavelength resonators, distributed mixer/amplifiers, evanescent wave amplification, super lens, *etc*. [4,5,6,7]. However, when frequency is higher, losses in materials (conductor and dielectric) will inevitably increase. Moreover, lumped elements are essential components for this kind of transmission lines. At higher frequencies (>a few GHz), high quality lumped elements (especially inductors) are rare and limited in their achievable values. In addition, although for a balanced CRLH-TL [7], the characteristic impedance is independent of frequency, its propagation constant remains dispersive.

Utilization of active devices with gain may be an ideal solution for compensating loss and obtaining more bandwidth for metamaterials. Several recent theoretical studies have discussed metamaterials incorporating gain devices or media for loss compensation and bandwidth enhancement [8,9]. Various experimental efforts, including adding amplifiers between the unit cells of CRLH-TL [10], utilizing parametric amplification of nonlinear circuit [11,12], including amplifier and phase shifter in magnetic metamaterial [13] and incorporating dye as gain medium in optical metamaterial [14], have been reported. However, active left-handed metamaterial with gain has yet to be widely achieved and used in applications due to design and fabrication complexities. For example, some fundamental physical properties, including the sign of the index of refraction of active metamaterials, are still not clear and conflicting conclusions have been reported [15,16,17,18].

In this paper, we present a simple and straightforward means of designing active transmission line metamaterials by incorporating an ideal gain device such as constant negative resistance or conductance into the unit cell of CRLH-TL. Various CRLH-TL designs with different positive and negative resistances/conductances and other realistic distributed unit cell parameters (series and shunt capacitances and inductances) are investigated. The complex propagation constants *γ* (*γ = α + jβ*, where *α* is the attenuation constant and *β* is the phase constant) are calculated using the transmission line equivalent circuit model approach [7]. The sign of the propagation constant (*α* and *β*) is determined by an alternative method for the active CRLH-TL metamaterials using a sign function which is based on the S-parameter evaluation of net gain (or loss). It is shown that by adding a negative resistance/conductance component into the unit cell of a CRLH-TL, left-handed phase constant *β* of the transmission line can be preserved while gain (negative *α*) is achieved. Possible implementation of negative resistance with semiconductor devices such as transistors and diodes and practical challenging issues are discussed.

## 2. Equivalent Circuit Model of Active CRLH-TL Metamaterials

**Figure 1:**
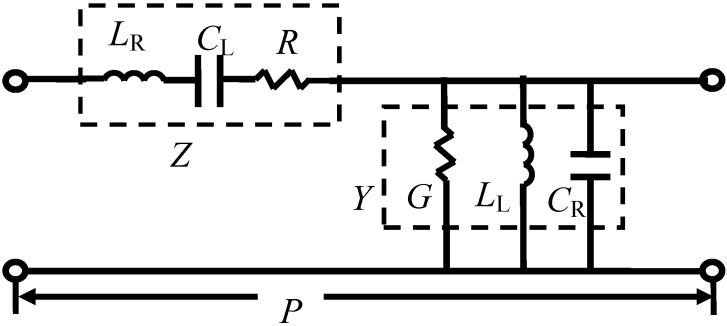
Equivalent circuit model of a unit cell of a general metamaterial CRLH-TL (active scenario: negative *R* and/or negative *G*).

The equivalent circuit model of a general metamaterial CRLH-TL unit cell is shown in Figure 1. The circuit parameters are: series inductance *L*_R_ with unit of *H/m*, series capacitance *C*_L_ with unit of *F∙m*, series resistance *R* with unit of *Ω/m*, shunt conductance *G* with unit of *S/m*, shunt inductance *L*_L_ with unit of *H∙m* and shunt capacitance *C*_R_ with unit of F*/m*. The subscripts R and L stand for RH and LH components, respectively. In the passive scenario, the resistance and conductance represent conductor and dielectric losses in the transmission line. However, an active device, for example, a negative resistance *R* (or negative conductance *G*) may be used to compensate the losses or even provide gain for the CRLH-TL.

As labeled in Figure 1, the series impedance *Z* and shunt admittance *Y* for the unit cell of the CRLH-TL are given by

(1) $$Z=R+j\left(\omega L_{R}-\frac{1}{\omega C_{L}}\right)$$

(2) $$Y=G+j\left(\omega C_{R}-\frac{1}{\omega L_{L}}\right)$$

Assuming homogeneity (the unit cell length p needs to satisfy p << λg/4, where λg is the guided wavelength) [4,5,6,7], the propagation constant can be written as

(3) $$\gamma p=(\alpha +j\beta )p=\pm \sqrt{ZY}\equiv \sqrt{a+jb}$$

in which *a* and *b* are the real and imaginary parts of the product of *Z* and *Y*, respectively. For a passive/lossless CRLH-TL, the sign choice of *α* and *β* have been discussed extensively [7]. However, for an active metamaterial, the sign choice has been a topic of debate [15,16,17,18]. Here we propose a sign function *S^’^(ω)* defined as,

(4) $$S^{\prime }\left(\omega \right)=+1,\;\text{if}\;1-({\left|S_{11}\right|}^{2}+{\left|S_{21}\right|}^{2})>0$$

(5) $$S^{\prime }\left(\omega \right)=-1,\;\text{if}\;1-({\left|S_{11}\right|}^{2}+{\left|S_{21}\right|}^{2})<0$$

where *S*_11_ and *S*_21_ are S-Matrix elements of the unit cell equivalent circuit. Then the attenuation constant *α*, phase constant *β*, effective refractive index *n*_eff_, effective permittivity *ε*_eff_ and effective permeability *µ*_eff_ can be obtained as the following,

(6) $$\alpha =S^{\prime }\left(\omega \right)\sqrt{\frac{a+\sqrt{a^{2}+b^{2}}}{2}}$$

(7) $$\beta =\frac{b}{2\alpha }$$

(8) $$n_{eff}=\frac{\gamma }{jk_{0}}=\frac{b}{2k_{0}\alpha }-j\frac{\alpha }{k_{0}}$$

(9) $$\varepsilon_{eff}=Y/(j\omega )$$

(10) $$\mu_{eff}=Z/\left(j\omega \right)$$

where *k*_0_ is the free space wave number.

It is worth mentioning that this method for determining the sign of the propagation constant is quite convenient for most of the cases. For lossless case such that *R = 0* and *G = 0*, obviously, Equations (7) and (8) are not available for the denominator being zero. However, this ideal lossless situation has been widely discussed in the literature [7].

## 3. Examples of Active CRLH-TL Metamaterial with Ideal Gain Devices

**Figure 2:**
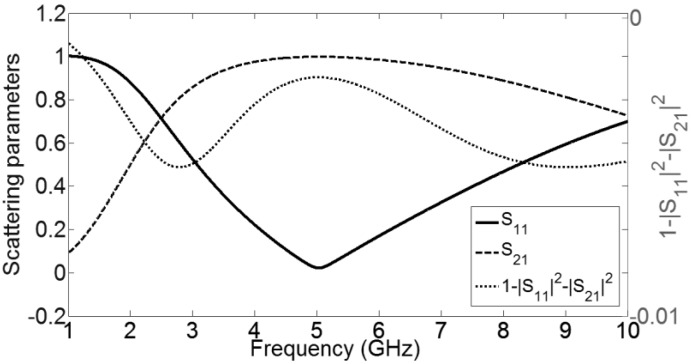
Scattering parameters and sign function of the active CRLH-TL unit cell.

As an example, a balanced passive CRLH-TL is first considered. The lumped element parameters are chosen to be: *L*_R_ = 1 *nH*, *C*_L_ = 1 *pF*, *L*_L_ = 1 *nH*, *C*_R_ = 1 *pF*, in which case the transition (zero-th order resonance) frequency is $f_{\Gamma }=\omega_{\Gamma }/\left(2\pi \right)=1/\left(2\pi \sqrt{L_{R}C_{L}}\right)=1/\left(2\pi \sqrt{C_{R}L_{L}}\right)=5GHz$ [7]. This lossless CRLH-TL is left-handed below 5 GHz with *β* *< 0* and right-handed above 5 GHz with *β* *> 0* (see Figure 4(b)). Now consider that an ideal negative resistance *R =* −1 *Ω* is introduced in the unit cell (see Figure 1) and for simplicity *G* = 0 is assumed. The S-parameters of the active unit-cell are plotted in Figure 2. As expected, the sign function for *α* in Equation (6) is negative because of the ideal gain element.

**Figure 3:**
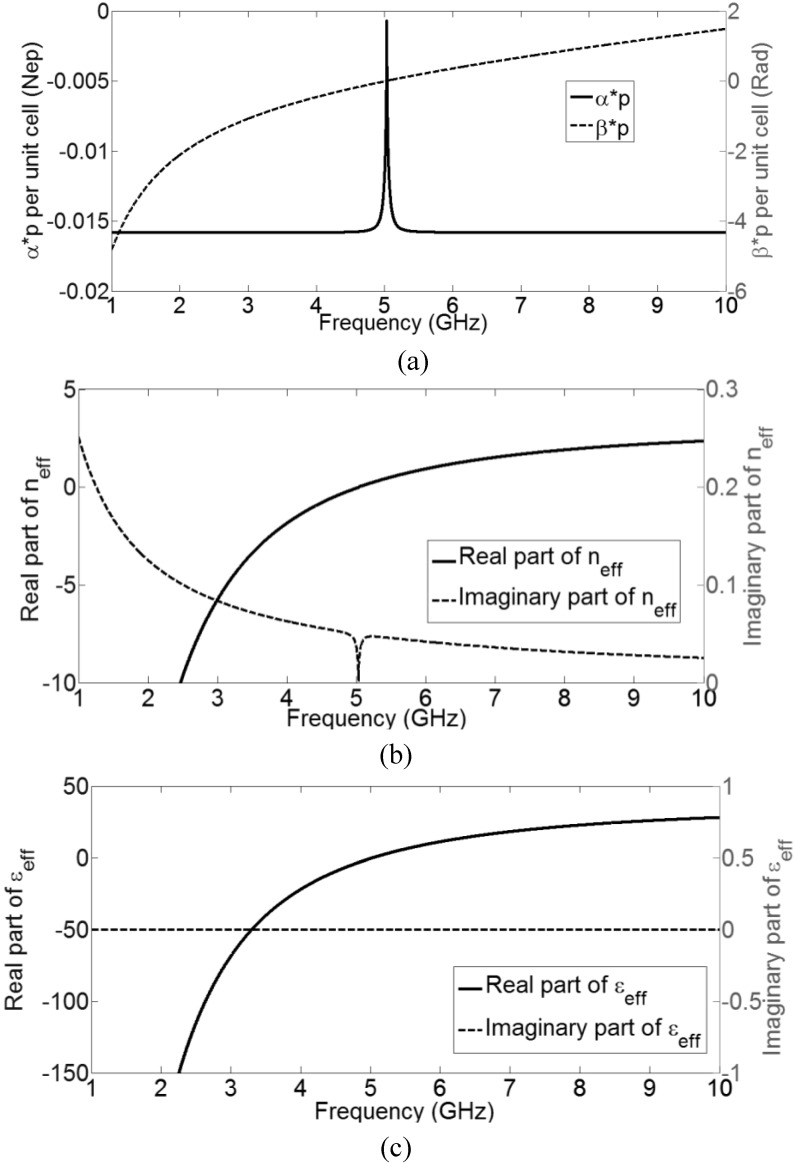

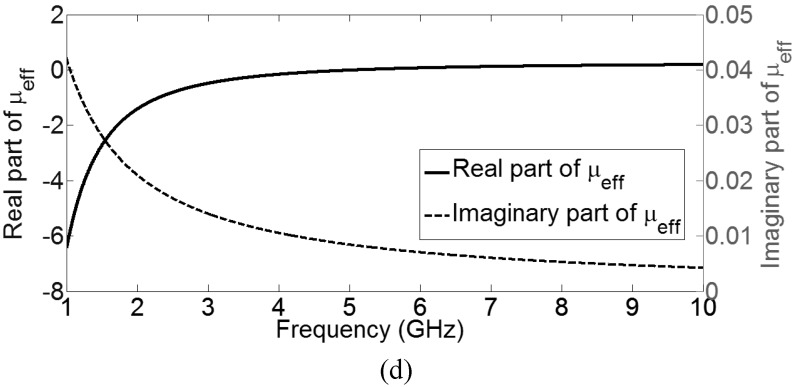
Parameters of the active CRLH-TL metamaterial as a function of frequency: (**a**) Attenuation constant *α* (solid line) and phase constant *β* (dotted line); (**b**) Real and imaginary part of the effective index of refraction *n*_eff_ (assuming a realistic unit cell physical length of 3 mm); (**c**) Real and imaginary part of the effective permittivity *ε*_eff_ (assuming a realistic unit cell physical length of 3 mm); (**d**) Real and imaginary part of the effective permeability *µ*_eff_ (assuming a realistic unit cell physical length of 3 mm).

Using Equations. (6)–(10), the complex propagation constant (*γp = αp + j βp*) and the effective material parameters (*n*_eff_, *ε*_eff_ and *µ*_eff_), as a function of frequency, can be calculated, as plotted in Figure 3. It should be noted that the results are valid only in regions for which the homogeneity assumption holds (*p << λ*_g_/4, or *βp <<* *π/2*). The complex propagation constant can also be extracted by using the ABCD matrix of the unit cell [7] (very similar results are yielded, not shown here).

It can be observed in Figure 3(a) that below 5 GHz, the phase constant *β* is negative, and above 5 GHz, the phase constant *β* is positive, just as the lossless CRLH-TL without the negative resistance. Moreover, the attenuation constant *α* is negative for all frequencies, indicating that this active transmission line not only maintains its composite right- and left-handed properties but also has gain. By assuming a reasonable physical size of the unit cell, for example, let *p =* 3 mm, the effective material parameters are calculated. The effective index of refraction *n*_eff_ of this active CRLH-TL (Figure 3(b)) has a negative and positive real part below and above 5 GHz, respectively, while its imaginary part is always positive, indicating gain. The effective permittivity *ε*_eff_ and permeability *µ*_eff_ are plotted in Figure 3(c) and 3(d), respectively. Both real parts of *ε*_eff_ and permeability *µ*_eff_ are negative below 5 GHz and positive above 5 GHz. The activeness or gain of this CRLH-TL is apparent in the positive imaginary part of *µ*_eff_, which is a direct consequence of the incorporation of the ideal negative resistance *R* in the unit cell. Therefore, this active CRLH-TL corresponds to a double negative (DNG) medium with gain in the magnetic constituent. Negative conductance *G* can also be incorporated to provide an active dielectric constituent.

**Figure 4:**
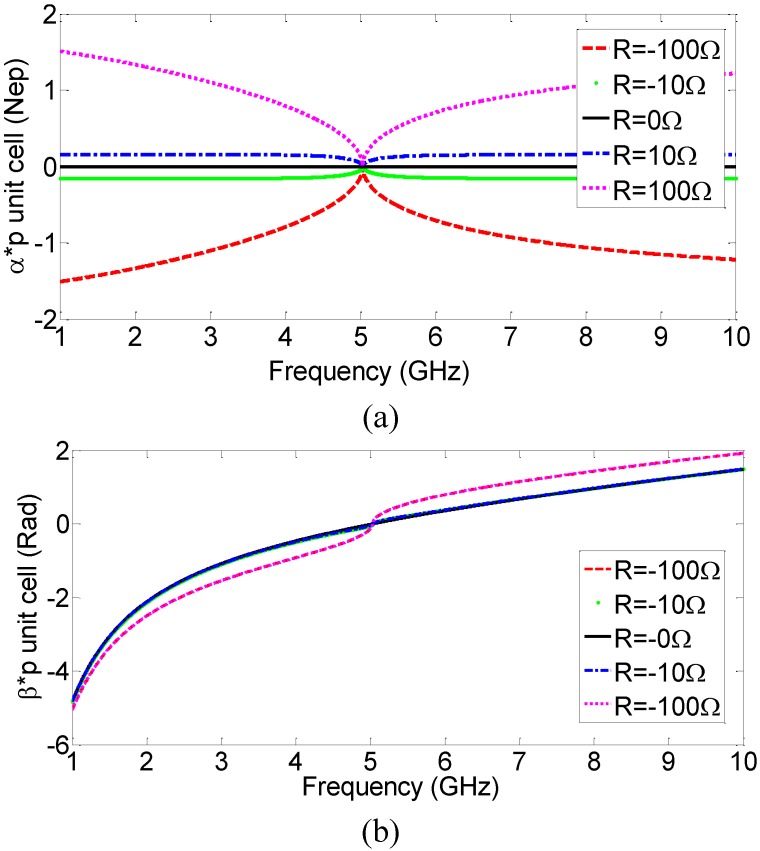
(**a**) Attenuation constant *α* and (**b**) phase constant *β*, of several CRLH-TLs with different series resistance values.

**Figure 5:**
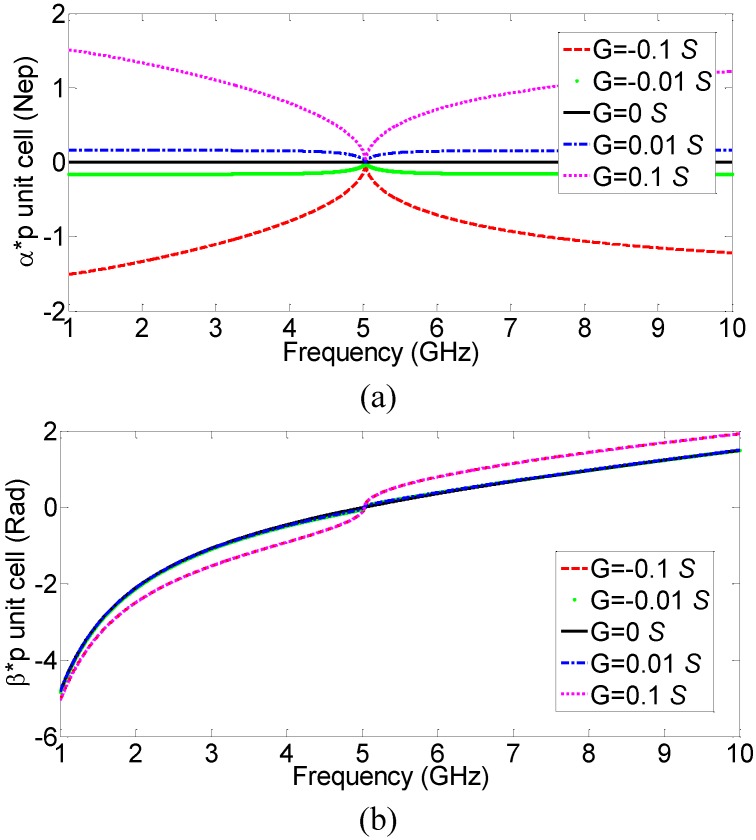
(**a**) Attenuation constant *α* and (**b**) phase constant *β*, of several CRLH-TLs with different shunt conductance values.

To evaluate the effect of the actual value of the series resistance *R* in Figure 1, the calculated propagation constants of several similar CRLH-TL with different *R* (active cases: *R =* −100 Ω and −10 Ω; lossless case: *R =* 0; and passive cases: *R =* 10 Ω, 100 Ω) are plotted in Figure 4. As expected, for negative resistance, the attenuation constant *α* (Figure 4(a)) is negative, indicating gain, and for positive resistance, the attenuation constant *α* is positive, indicating loss. The phase constant *β*, on the other hand, is qualitatively the same for all the different series resistance values, demonstrating that the choice of *R* does not affect the handedness of the transmission line. Various corresponding negative conductances are also evaluated and active CRLH-TL with similar properties, except being dielectrically active, is obtained, as shown in Figure 5.

## 4. Possible Implementation and Challenges of Active CRLH-TL

To realize in practice the proposed active CRLH-TL metamaterial at microwave frequencies, negative resistance and/or conductance using semiconductor transistors or diodes will be ideal as the gain devices. For example, it is well known that negative resistance devices made of transistors or certain diodes have been used to compensate losses in active microwave filters and increase bandwidth and gain in distributed amplifiers [19,20,21,22,23], in which case DC power supplies serve as the energy source. Potentially suitable semiconductor device technologies for active microwave metamaterials include GaAs PHEMT (Pseudomorphic High Electron Mobility Transistors), InP HEMT, InP HBT (heterojunction bipolar transistors), silicon CMOS, SiGe BiCMOS, various RTD (resonant tunneling diodes) and GaAs Gunn diodes (all of them can operate up to 100 GHz or even higher frequency) [24,25]. Furthermore, potential next generation nano devices including carbon nanotube (CNT) junctions [26] and graphene based devices may also be promising [27].

Several important issues need to be considered when implementing gain devices into the proposed CRLH-TL. First, parasitic capacitances and inductances exist in active devices and their impacts increase with frequency. Therefore, ideal frequency independent negative resistance is not possible to realize. Care must be taken to account for all the parasitic effects or preferably absorb at least some of the parasitics into the metamaterial transmission line design. One important design criteria is that the negative resistance used should be bilateral to simplify the design process. Second, unlike the ideal model discussed in the previous section, all active gain devices are intrinsically nonlinear so that nonlinear effects such as power dependence, higher order harmonics, intermodulation, *etc*., need to be considered when designing and modeling the properties of active metamaterials. On the other hand, nonlinearity brings opportunity as well as complexity. For example, frequency conversion applications such as mixing and multiplying may be realized. The third challenging issue is the stability of active metamaterials. Maintaining stable operation of active circuits may be critical as they are known to be very prone to unwanted oscillations.

**Figure 6:**
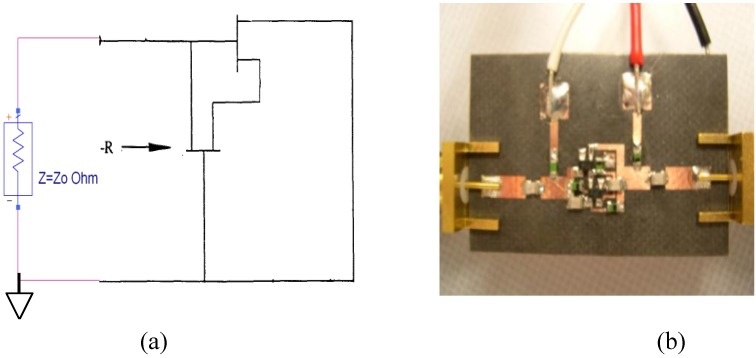
(**a**) Simplified schematic of a negative resistance circuit using a pair of transistors; (**b**) Fabricated negative resistance realization using GaAs PHEMT devices in a microstrip test fixture.

**Figure 7:**
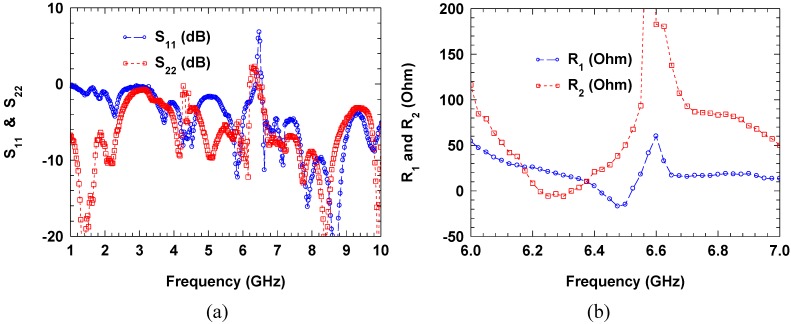
(**a**) Measured one-port S-parameters (circles are the reflection coefficients with port 2 grounded; and squares are the reflection coefficients with port 1 grounded) of the negative resistance circuit using a pair of transistors; (**b**) Calculated equivalent resistances from the measured one-port S-parameters (circles are the resistance seen at port 1 with port 2 grounded; and squares are the resistance seen at port 2 with port 1 grounded).

As a proof-of-concept, a negative resistance circuit consisting of a pair of GaAs PHEMT devices with reactive load which functions as a feedback element is studied. Figure 6 show the simplified schematic of a transistor-pair based negative resistance circuit and its GaAs PHEMT implementation in a test fixture. The transistor used here is a commercial off-the-shelf part, ATF-551M43, which can provide gain up to 20 GHz. The reflection coefficients at a particular bias setting (*V*_ds_ = 3 V and *V*_gs_ = 0.3 V) of this circuit with one of the ports shorted to ground are measured using a vector network analyzer. The measured one-port reflection coefficients are plotted in Figure 7(a), in which *S*_11_ and *S*_22_ represent port 2 and port 1 being grounded, respectively. It can be observed that the measured reflection coefficients are greater than 0 dB near 6.4 GHz. The equivalent resistances are calculated from the measured data and plotted in Figure 7(b), in which *R*_1_ and *R*_2_ represent port 2 and port 1 being grounded, respectively. Although negative resistance is measured for the fabricated circuit, there are some issues that need to be addressed for practical insertion into metamaterial unit cells. For example, as shown in Figure 7, the measured response of the fabricated negative resistance circuit is not exactly reciprocal as designed, likely due to imperfections in the fabrication process and lumped elements used. More importantly, applying the four DC bias voltages for the transistor pair requires quite complicated DC blocking and RF isolation scheme, which significantly increases the circuit size and complexity and limits the useful negative resistance bandwidth. Nevertheless, with potential implementation utilizing advanced integrated circuit technologies (such as CMOS), most of those issues can be solved and it is feasible to achieve active metamaterial with this kind of gain devices incorporated at the unit cell level.

## 5. Conclusions

This paper presents a straightforward method to design active CRLH-TL metamaterials. Several examples with different value of negative resistance/conductance are shown. It is demonstrated that by incorporating ideal gain devices at the unit cell level of a CRLH-TL, the negative phase constant of the transmission line can be maintained (*i.e*., left-handedness kept) while gain is obtained (*i.e*., negative attenuation constant). Possible implementation of this kind of active CRLH-TL and several design challenges are also discussed. Active metamaterials incorporating gain have rich physics to be further explored due to their intrinsic nonlinearity and other complexities. The potential to compensate loss/realize gain and increase bandwidth may provide a new paradigm for bringing many future applications into reality. As simple as the active CRLH-TL discussed here, it will be very useful not only for designing and achieving new and high performance microwave circuits and antennas, but also as a model system to study fundamental physics of active metamaterials in general and shed light on metamaterial applications at other spectra including optical frequencies.
